# The RIN4-like/NOI proteins NOI10 and NOI11 modulate the response to biotic stresses mediated by RIN4 in Arabidopsis

**DOI:** 10.1007/s00299-024-03151-9

**Published:** 2024-02-15

**Authors:** Estefania Contreras, Manuel Martinez

**Affiliations:** 1 Centro de Biotecnología y Genómica de Plantas (CBGP), Universidad Politécnica de Madrid (UPM)-Instituto Nacional de Investigación y Tecnología Agraria y Alimentaria (INIA/CSIC), Campus de Montegancedo, 20223 Madrid, Spain; 2 Departamento de Biotecnología-Biología Vegetal, Escuela Técnica Superior de Ingeniería Agronómica, Alimentaria y de Biosistemas, UPM, Madrid, Spain

**Keywords:** Arabidopsis, Biotic stress, Plant defense, *Pseudomonas syringae*, RIN4-like/NOI proteins, *Tetranychus urticae*

## Abstract

**Key message:**

NOI10 and NOI11 are two RIN4-like/NOI proteins that participate in the immune response of the Arabidopsis plant and affect the RIN4-regulated mechanisms involving the R-proteins RPM1 and RPS2.

**Abstract:**

The immune response in plants depends on the regulation of signaling pathways triggered by pathogens and herbivores. RIN4, a protein of the RIN4-like/NOI family, is considered to be a central immune signal in the interactions of plants and pathogens. In *Arabidopsis thaliana*, four of the 15 members of the RIN4-like/NOI family (*NOI3*, *NOI5*, *NOI10*, and *NOI11*) were induced in response to the plant herbivore *Tetranychus urticae*. While overexpressing *NOI10* and *NOI11* plants did not affect mite performance, opposite callose accumulation patterns were observed when compared to *RIN4* overexpressing plants. In vitro and in vivo analyses demonstrated the interaction of NOI10 and NOI11 with the RIN4 interactors RPM1, RPS2, and RIPK, suggesting a role in the context of the RIN4-regulated immune response. Transient expression experiments in *Nicotiana benthamiana* evidenced that NOI10 and NOI11 differed from RIN4 in their functionality. Furthermore, overexpressing *NOI10* and *NOI11* plants had significant differences in susceptibility with WT and overexpressing *RIN4* plants when challenged with *Pseudomonas syringae* bacteria expressing the AvrRpt2 or the AvrRpm1 effectors. These results demonstrate the participation of NOI10 and NOI11 in the RIN4-mediated pathway. Whereas RIN4 is considered a guardee protein, NOI10 and NOI11 could act as decoys to modulate the concerted activity of effectors and R-proteins.

**Supplementary Information:**

The online version contains supplementary material available at 10.1007/s00299-024-03151-9.

## Introduction

Regulation of signaling in response to pathogens and herbivores is critical for a suitable immune response in plants. Two interconnected immune systems are activated through the recognition of foreign molecules by specific cell-surface or intracellular receptors (Delplace et al. 2022). The first system of plant immunity involves plant cell-surface receptors or pattern recognition receptors (PRR) that recognize highly conserved molecules called microbe-, pathogen- or herbivore-associated molecular patterns (MAMPs, PAMPs, or HAMPs), activating the PAMP-triggered immunity (PTI). This type of immunity can be suppressed by effector molecules produced by the invader organism. Well-studied examples are some bacteria avirulence factors (Avr), which modify host proteins to manipulate PTI. The second system of plant immunity involves the recognition of these effectors by the host disease resistance proteins (R-proteins) that activate the effector-triggered immunity (ETI), associated with programmed cell death at the site of infection to limit pathogen progression, also referred to as hypersensitive response (HR) (Bigeard et al. 2015; Jones and Dangl 2006). Some Avr factors directly interact and posttranslationally modify R-proteins, triggering ETI defense responses, but in some cases, R-proteins respond to the modification of another host protein that is “guardee” by these R-proteins (Van der Biezen and Jones 1998).

RIN4 (RPM1-interacting protein 4) has been deeply studied in the context of *Pseudomonas syringae* infection and is considered a “guardee” protein that can regulate both branches of the plant immune system, PTI and ETI, in *Arabidopsis thaliana* (Ray et al. 2019; Zhao et al. 2021). It behaves as a negative regulator of the PTI (Kim et al. 2005) and is also a target of multiple Avr factors from *P. syringae* such as AvrB, AvrRpm1, or AvrRpt2 triggering ETI (Axtell and Staskawicz 2003; Mackey et al. 2002). AvrB and AvrRpm1 induce hyperphosphorylation of RIN4 by RIPK (RPM1-induced protein kinase) and other related RLCK (plant receptor-like cytoplasmic kinases), and the phosphorylation in T166 is recognized by the R protein RPM1 promoting RPM1-mediated ETI (Liu et al. 2011). In addition, AvrRpt2 causes RIN4 elimination resulting in the activation of the R protein RPS2 (Axtell and Staskawicz 2003).

Because of its highly disordered structure, RIN4 is considered a central immune signal that serves as a hub for protein complex formation. Besides interactions with the R-proteins RPM1 and RPS2, RIN4 contributes to plant immunity by interacting with other proteins. RIN4 is involved in the control of stomata opening by its interaction and positive regulation of the H^+^-ATPases AHA1 and AHA2 (Lee et al. 2015). Likewise, its interaction with the exocyst complex subunits EXO70B1 and EXO70E2 suggests a role in the regulation of callose deposition (Redditt et al. 2019). These findings support the relevance of disordered proteins such as RIN4 in the connection of immune-related pathways.

Besides RIN4, other 14 RIN4-like/NOI (NO_3_-induced) genes are present in the genome of Arabidopsis (Afzal et al. 2013; Sun et al. 2014). Apart from comparative and evolutionary sequence-based characterizations, scarce information has been reported on the functional relevance of these RIN4-like/NOI family members. In previous studies, we identified that four Arabidopsis proteins from the RIN4-like/NOI protein family were overexpressed after infestation by the spider mite *Tetranychus urticae*, and were also differentially regulated after the feeding of other arthropod species (Contreras and Martinez 2022). Two of these proteins (NOI10 and NOI11), as RIN4, have two conserved NOI domains, and the other two proteins (NOI3 and NOI5) are shorter in length and have only one. Interestingly, the NOI10 and NOI11 proteins differ from RIN4 in the conservation of posttranslationally modified residues, the structure of the C-NOI domain, and the predicted binding motifs in the disordered regions. These results suggested that RIN4-like/NOI members might have novel functions different from those assigned to RIN4, likely involving adaptation to stress specialization (Contreras and Martinez 2022).

In this work, we provide functional data of mite-induced RIN4-like/NOI proteins. We demonstrate that NOI10 and NOI11 participate in the immune response of the Arabidopsis plant and affect the RIN4-regulated mechanisms involving the R-proteins RPM1 and RPS2.

## Materials and methods

### Plant material and growth conditions

*A. thaliana* Columbia (Col-0) ecotype was used as wild-type. *A. thaliana* T-DNA mutant lines were obtained from the Nottingham Arabidopsis Stock Centre (NASC). Seeds were sterilized with 70% ethanol for 2 min and with 5% (V/V) sodium hypochlorite + 5% (W/V) SDS for 10 min, and then washed with sterile distilled water. For soil growth, a mixture of peat moss and vermiculite (3:2 v/v) was used. Sterilized seeds were stratified in darkness at 4 °C for 5 days. Plants were grown in growth chambers (Sanyo MLR-351-H) under controlled conditions (23 °C ± 1 °C, > 70% relative humidity, and a 16 h/8 h day/night photoperiod). *Nicotiana benthamiana* plants were grown in a greenhouse at 22 °C ± 1 °C, > 70% relative humidity, and a 16 h/8 h day/night photoperiod for 3 weeks before infiltration.

To generate overexpression lines, *RIN4/NOI* cDNAs were amplified from *A. thaliana* Col-0 cDNA by PCR (Bio-Rad T100 Thermal Cycler) using specific primers (Supplementary Dataset S1). DNA fragments were excised from agarose gels, purified using Qiaex II Gel Extraction Kit (Qiagen), and cloned into the pENTR1A vector. RIN4/NOI genes were then carried to the pGWB2 (CaMV35S, no tag) Gateway binary vector (Nakagawa et al. 2007) by LR reaction (Gateway LR clonase II enzyme mix, Thermo Fisher Scientific). Plasmids were transformed by electroporation into *Agrobacterium tumefaciens* C58C1 strain using an ECM 630 Electroporator (BTX). The recombinant plasmids were introduced into *A. thaliana* Col-0 plants using *Agrobacterium* floral dip transformation (Clough and Bent 1998). Shoots were regenerated on a selective medium containing hygromycin (40 mg/L) and plants were self-fertilized until homozygous lines were identified. Homozygous plants with the highest transgene expression levels coming from different transformation events were selected for further experiments.

### Spider mite maintenance and infestation

*T. urticae* London strain (Acari: Tetranychidae), provided by Dr. Miodrag Grbic (UWO, Canada), was reared in bean plants (*Phaseolus vulgaris*) in growth chambers (Sanyo MLR-350-H) at 25 °C ± 1 °C and a 16 h/8 h day/night photoperiod. For *A. thaliana* infestation, twenty *T. urticae* female adults per plant were placed on the leaf surface using a brush, and infested plants were kept in the same conditions as the mite colony until collected.

### Plant damage determination

To quantify leaf damage, 2-week-old rosettes were infested with 20 T*. urticae* female adults per plant for 4 days. Images for damage quantification were recovered using an HP Scanjet (HP Scanjet 5590 Digital Flatbed Scanner series). The total and damaged area of each rosette was measured using Adobe Photoshop CS software and analyzed using Ilastik and Fiji, as previously described (Ojeda-Martinez et al. 2020). Chlorotic spots were quantified as leaf damage areas. Eight biological replicates from independent rosettes were used for each genotype.

For electrolyte leakage and callose assays, 10 mites were placed onto 1 cm diameter leaf disks for 24 h. To evaluate callose deposition, leaves were incubated in 95% (V/V) ethanol and stained with aniline blue (Sánchez-Vallet et al. 2012). Images were visualized using the Leica MZ10F fluorescence stereoscope. Ten biological replicates from independent rosettes were used for each genotype. For electrolyte leakage measurements, mite-infested and control leaf disks (6 per replicate) were incubated in 4 ml distilled water for 4 h at 32 °C in a water bath. Electrolyte measurements were performed with a SensION + EC7 (Hach) conductimeter. Total electrolyte content was determined similarly after boiling the samples for 20 min at 100 °C. Electrolyte leakage of the samples was represented as the percentage of the total electrolyte content.

### Determination of gene expression

The presence and homozygous status of the T-DNA insertion lines were validated by conventional PCR (Bio-Rad) using specific primers designed through the Salk Institute website. Primer sequences are indicated in Supplementary Dataset S1. The genomic DNA used for conventional PCR was isolated from Arabidopsis T-DNA insertion and WT lines basically as described (Sambrook et al. 2001).

The expression of the *RIN4*/*NOI* genes was determined to check transcript accumulation in *A. thaliana* overexpressing and T-DNA insertion lines. The expression of these genes was also assessed at different time points after spider mite feeding to validate RNAseq results and after 3 h in *noi10* lines for compensation assays. Three biological replicates were used in each experiment. Six *A. thaliana* rosettes were pooled for each biological replicate, frozen in liquid nitrogen, and stored at − 80 ºC until use. Total RNA was extracted by the phenol/chloroform method, followed by precipitation with 8 M LiCl (Oñate-Sánchez and Vicente-Carbajosa 2008). Complementary DNAs (cDNAs) were synthesized from 2 μg of RNA using the Revert Aid™ H Minus First Strand cDNA Synthesis Kit (Thermo Scientific) following manufacturer instructions. Two RT-qPCR were performed for each biological replicate in a LightCycler 480 Software release 1.5.0 SP4 (Roche). Ubiquitin was used as the housekeeping gene for Arabidopsis. mRNA quantification was expressed as relative expression levels (2^−*dCt*^) or fold change (2^*−ddct*^) (Livak and Schmittgen 2001). Used primers are included in Supplementary Dataset S1.

### Transient expression in *N. benthamiana*

For transient expression assays, *RPM1*, *RPS2*, *RIPK*, *RIN4*, *NOI10,* and *NOI11* genes were amplified from *A. thaliana* Col-0 cDNA by PCR using specific primers (Supplementary Dataset S1). A C-terminus HA tag was added to RPM1, RPS2, and RIPK in three PCR amplification steps. *RIN4*/*NOI* genes were cloned into the pGWB5 (CaMV35S, C-terminus GFP tag) Gateway binary vectors as above described. *RPM1*, *RPS2*, and *RIPK* were transferred similarly to the pEAQ-HT-DEST3 vector. To generate RIN4/NOI phosphomimetic mutants, point mutations to RIN4 T166E, NOI10 T166E, and NOI11 T168E were introduced by PCR using primers containing the mutation (Supplementary Dataset S1).

*A. tumefaciens* carrying *RPM1*, *RPS2*, and *RIPK* in pEAQ-HT-DEST3 vector or *RIN4*/*NOI* genes in pGWB5 vector were grown in Luria–Bertani medium with rifampicin + kanamycin for 24 h. Cells were collected by centrifugation, resuspended in 10 mM MES, 10 mM MgCl_2_, 150 µM acetosyringone to the corresponding OD_600_ (0.3 for NOI genes, 0.5 for RPM1/RIPK, 0.2 for RPS2, and 0.9 for RIN4 when coexpressed with RIPK + RPM1) and incubated for 3 h RT. Cells were then infiltrated into the abaxial side of 3-week *N. benthamiana* leaves using needless syringes. For localization assays, transient expression of genes fused to the GFP tag was observed 3 d after infiltration using a Leica SP8 confocal microscope with 40 × magnification. For HR experiments, leaves infiltrated with RPS2 + RIN4/NOI proteins or RPM1 + RIN4/NOI phosphomimetic mutants were examined for macroscopic HR after 3 d, and those infiltrated with RPM1 + RIPK + RIN4/NOI proteins were examined after 5 d. All combinations were coexpressed with the P19 protein of tomato bushy stunt virus (TBSV) to enhance transient expression.

### Membrane Yeast Two-Hybrid (MYTH)

For Membrane Yeast Two-Hybrid (MYTH) experiments, proteins fused to split-ubiquitin Cub domain (baits) or Nub domain (preys) were obtained. To construct baits, *RIPK* was cloned into the pAMBV4 vector (strong ADH1 promoter), and *RPM1*, *RPS2*, and *RIN4*/*NOI* genes were cloned into the pTLB-1 vector (strong TEF promoter). *RIN4* was also cloned in pBT3-N (weak CYC1 promoter). To construct the prey, *RIPK* was cloned into pPR3-N (ADH1 promoter) and *RPM1*, *RPS2*, and *RIN4*/*NOI* genes into the pPR3-N vector (CYC promoter). Primers used to amplify the genes and facilitate homologous recombination are included in Supplementary Dataset S1. Vectors were introduced in *Saccharomyces cerevisiae* DY1457 strain by the Li-Acetate transformation method (Schiestl and Gietz 1989) employing homologous recombination (Iyer et al. 2005; Snider et al. 2010). DOB + auxotrophic selection media CSM-L (without leucine) and CSM-W (without tryptophan) (Bioworld) were used to select positive yeast colonies harboring baits and preys, respectively. Plasmids were recovered by phenol:chloroform method and introduced into *Escherichia coli* DH5α by electroporation for gene validation and sequencing. *S. cerevisiae* NMY51 strain was transformed with the different combinations of baits and preys, and positive colonies were selected in DOB+CSM-L-W. Selected colonies were grown and 7 µL of the cultures were dropped onto solid DOB+CSM-L-W-H with different concentrations of 3-AT (3-amino-1,2,4-triazole) (Merck).

### Co-immunoprecipitation assays

*N. benthamiana* leaves coinfiltrated with *A. tumefaciens* expressing HA-tagged RPM1, RIPK, or RPS2 and RIN4/NOI proteins fused to GFP tag were collected after 3 d and ground in liquid nitrogen. Two hundred mg of ground tissue was homogenized in 5 ml CoIP buffer (50 mM Tris HCl, 125 mM NaCl, 0.1% tween-20, 1 mM DTT, 10% glycerol, 1 mM PMSF, complete EDTA-free protease inhibitor cocktail (Merck), pH 7.4). The lysate was centrifuged at 13,000 rpm 15 min 4 °C. The supernatant (input) was incubated overnight at 4 °C in agitation with Dynabeads protein G (Thermo Fisher Scientific) previously bound to anti-HA high-affinity antibody (Merck) according to the manufacturer’s protocol. Protein bound to the bead complex was pulled from the extract using a DynaMag-2 magnet, washed 3 times with CoIP buffer, and analyzed by western blot. The complex was transferred to an Amersham Protran nitrocellulose membrane (GE Healthcare) and immunodetected with anti-HA high-affinity antibody (Merck) (1:1000) followed with anti-rat IgG coupled to peroxidase (1:15,000) (Merck) to detect the Immunoprecipitate (IP) or with a monoclonal anti-GFP antibody (Miltenyi Biotec) to detect the Co-Immunoprecipitate (CoIP). Chemiluminescent detection was performed in an iBright Imaging System (Thermo Fisher Scientific) using ECL select WB reagent (GE Healthcare).

### Bacterial infection assays

*P. syringae* pv. tomato DC3000 genotypes, provided by Dr. Emilia Lopez-Solanilla (CBGP, UPM-INIA/CSIC, Spain), were grown in solid King’s B (KB) medium (King et al. 1954) for 24 h and in solid KB + 25 μg/ml rifampicin for 48 h. Bacterial cells were collected, washed twice in 10 mM MgCl_2_, diluted to 10^8^ CFU/ml for spray inoculations on 3-week-old Arabidopsis plants, and applied with 5 spray pulses per plant. Infected plants were kept in growth chambers at 23 °C and > 70% humidity. Bacterial growth in planta was determined 4 days post-inoculation (dpi) by collecting samples from leaves with a 6 mm diameter cork borer. Three leaf disks from the same plant were pooled and homogenized in 10 mM MgCl_2_ and serial dilutions were dropped onto KB plates for colony-forming unit (CFU) counting. CFU counting was used as an indicator of plant susceptibility.

### In silico analyses

Venn diagrams were performed using the InteractiVenn tool (Heberle et al. 2015). The 100 genes with the highest expression correlation with *RIN4*, *NOI10*, and *NOI11* were obtained from the ATTED II database (Obayashi et al. 2022). Gene enrichment analyses were performed with the Bonferroni step-down test using the ClueGO package (Bindea et al. 2009) in Cytoscape (Shannon et al. 2013). STRING database version 11.5 (Szklarczyk et al. 2019) was used to construct the RIN4-centered protein–protein physical-based network. Transcriptomic data on *RIN4*, *NOI10*, and *NOI11* expression in response to *P. syringae* were extracted from the GEO database, accession numbers GSE45212 and GSE198022 (Edgar et al. 2002).

### Statistical analyses

To design a suitable statistical approach, each poll of data was previously subjected to normality and homoscedasticity tests. Then, one-way ANOVA was applied, followed by Duncan’s multiple comparison tests (*P* < 0.05). The statistical analysis was performed using Statgraphics v19. The number of replicates is shown in the figure legends.

## Results

### Mite-induced RIN4-like genes have low basal expression and are located in the plasma membrane

**Fig. 1:**
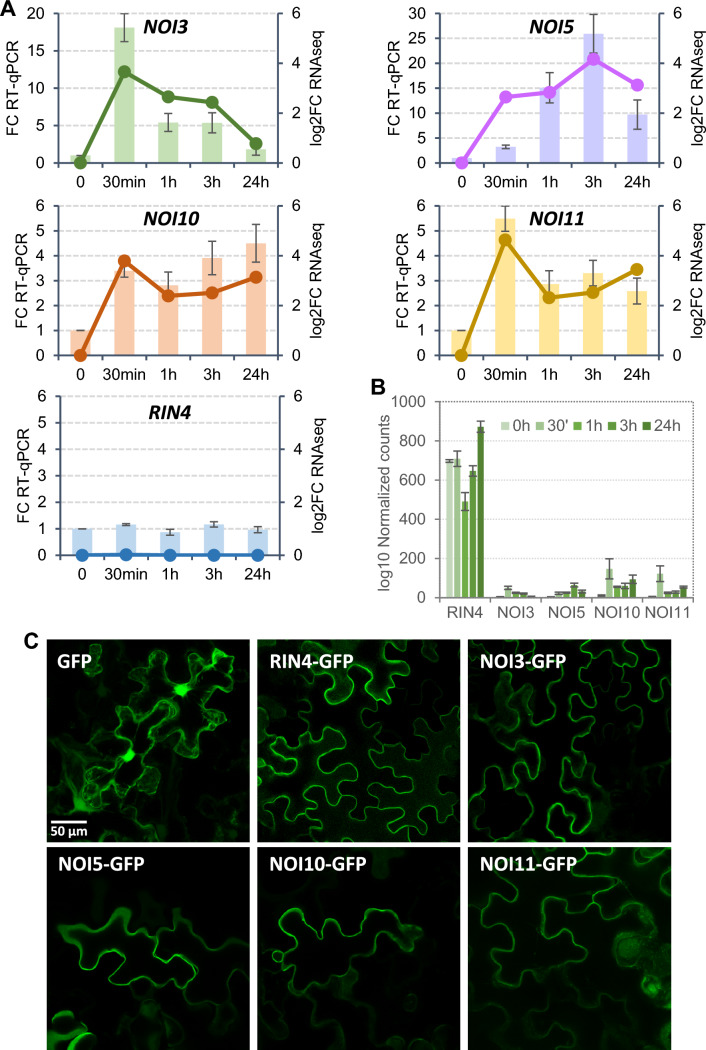
Gene expression after *T. urticae* infestation and subcellular location of RIN4-like/NOIs. (**A**) Correlation between RNAseq data (lines) and RT-qPCR validation (bars) in Arabidopsis for *NOI3*, *NOI5*, *NOI10*, *NOI11*, and *RIN4* after 30 min, 1 h, 3 h, and 24 h of mite infestation. Data are means of three biological replicates. (**B**) Log_10_ normalized counts of RIN4-like/NOIs according to RNAseq data (Santamaria et al. 2021). (**C**) Confocal images of *N. benthamiana* leaves transiently transformed with 35S::GFP or 35S::RIN4-like/NOIs-GFP constructs. A scale bar common for all images is shown

In a previous RNAseq analysis, four RIN4-like genes, *NOI3*, *NOI5*, *NOI10*, and *NOI11*, were identified as transcriptionally induced by infestation with the spider mite *T. urticae* in Arabidopsis unlike RIN4, whose expression remained constant (Contreras and Martinez 2022). Mite-induction of the four genes was corroborated by RT-qPCR analyses (Fig. 1A). Notably, strong variation in the basal expression of these genes was found when the number of normalized reads of the RNAseq data was analyzed in the original dataset (Santamaria et al. 2021). Whereas *RIN4* is constitutively expressed in Arabidopsis leaves, the expression of the four induced RIN4-like/NOI genes was very low before mite infestation (Fig. 1B).

Subcellular location was checked by transient expression of RIN4-like/NOI genes fused to GFP in *Nicotiana benthamiana* leaves. As for RIN4, the RIN4-like proteins have cysteine residues at the C-terminal end, which are predicted to be S-palmitoylated. The observation of the GFP fluorescence close to the plasma membrane confirmed similar subcellular locations for RIN4 and RIN4-like/NOI proteins (Fig. 1C).

### Overexpression of RIN4-like/NOI genes does not affect mite damage and, unlike RIN4, does not impair callose deposition

**Fig. 2:**
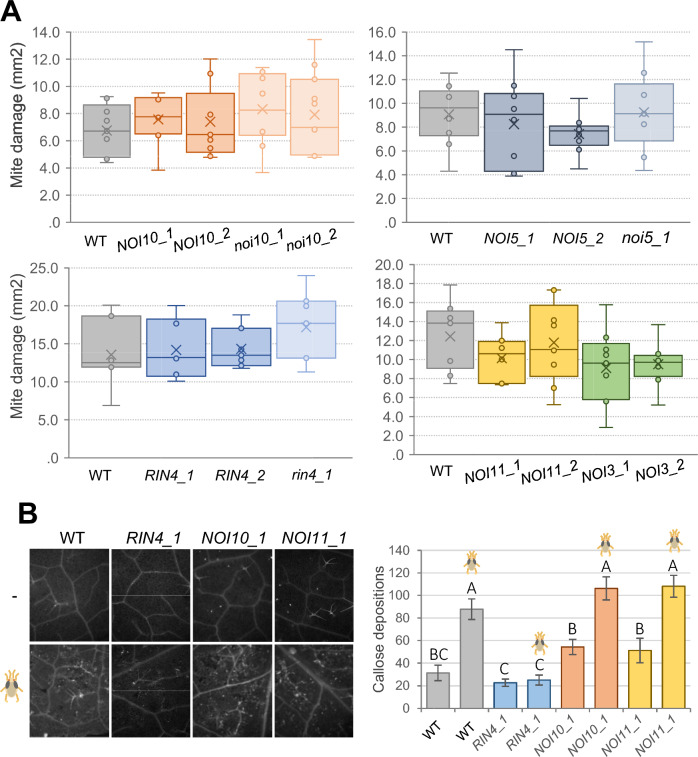
Plant damage and callose quantification after mite feeding on Arabidopsis RIN4-like/NOI genotypes. (**A**) The foliar damaged area was quantified after 4 d of mite infestation. Data are mean ± SE of ten replicates. (**B**) Callose deposition in leaves after 24 h of mite infestation. Representative images (left) and callose quantification (right). Data are mean ± SE of ten replicates. Different letters indicate significant differences (*P* < 0.05, One-way ANOVA followed by Duncan multiple comparisons test)

The upregulation of the RIN4-like/NOI genes in response to the mite attack suggests a role in plant defense. To test this possibility, we generated overexpressing (OE) Arabidopsis lines and selected two of these lines for each of the RIN4-like genes (Supplementary Fig. S1). Several T-DNA insertion lines that affect RIN4-like/NOI genes were selected from existing collections, but only two homozygous lines for *NOI10* and one for *NOI5* and *RIN4* showed a significantly reduced expression of the target genes (Supplementary Fig. S1). Homozygous overexpressing and T-DNA insertion lines, as well as the corresponding wild-type (WT) plants, were infested with spider mites, and plant damage was quantified after 4 days of mite feeding. No significant differences were found among the different genotypes (Fig. 2A). To analyze a possible RIN4-like/NOI function redundancy or an effect of gene compensation, the expression of *NOI3*, *NOI5*, and *NOI11* genes was assessed in the *noi10* T-DNA insertion lines and the WT plants. No significant differences were found in the expression of these genes, suggesting that the loss of function of NOI10 does not affect the expression of the other NOIs (Supplementary Fig. S2). Likewise, electrolyte leakage measurements before and after mite challenge did not provide any significant difference when compared in the WT plants and the OE or T-DNA insertion lines of *NOI5* and *NOI10* (Supplementary Fig. S3).

The absence of a leaf damage phenotype does not rule out other phenotypical alterations concerning plant defense. As callose deposition is an early phenotypical feature in the response to pathogens and herbivores, the callose accumulation in response to mite attack was analyzed in the *NOI10*, *NOI11*, and *RIN4* OE lines, as well as in WT plants. Upon mite infestation, *RIN4* OE lines showed a significantly lower accumulation of callose depositions than WT plants as expected for a negative regulator of PTI. Remarkably, unlike *RIN4* OE lines, the *NOI10* and *NOI11* OE lines behaved similarly to WT plants (Fig. 2B).

### *NOI10*,* NOI11*, and *RIN4* are coexpressed with different sets of defense-related genes

Since NOI10, NOI11, and RIN4 are the only three RIN4-like/NOI proteins in Arabidopsis with two-NOI domains, we decided to focus the analysis on them. Differences in gene expression and callose deposition after spider mite challenge point to dissimilar roles in biotic stress signaling.

**Fig. 3:**
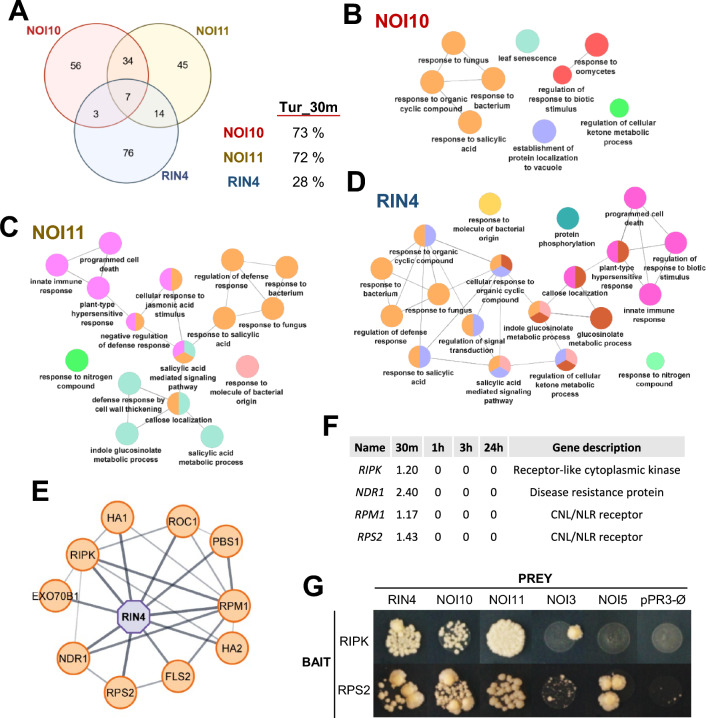
Gene coexpression and protein interaction analyses of RIN4-like/NOIs. (**A**) Venn diagram showing the number of specific and shared genes present in the lists of the 100 most coexpressed genes with *NOI10*, *NOI11*, and *RIN4* in the ATTED II database. The percentage of these genes induced at 30 min of *T. urticae* infestation is included. (**B–D**) Networks of the most enriched biological processes using the 100 most coexpressed genes with *NOI10* (**B**), *NOI11* (**C**), and *RIN4* (**D**), created with the ClueGO tool in Cytoscape. (**E**) Network of the physical interactors of RIN4 obtained in the STRING database and visualized in Cytoscape. (**F**) Differential gene expression (log2FC) of RIN4 interactors after *T. urticae* treatment according to RNAseq data (Santamaria et al. 2021). (**G**) MYTH results of protein–protein interaction using RIN4-like/NOIs as prey and RIN4 interactors as baits

To obtain information on their physiologic functions, the 100 genes with the highest correlated expression with each two-NOI gene were obtained from the ATTED II database (Supplementary Dataset S2). Of these 100 genes, only 7 genes were shared on the three lists and *NOI10*/*NOI11* was the combination with more common genes (Fig. 3A). Searches in the differentially expressed sets of genes from the original RNAseq analysis of the Arabidopsis response to *T. urticae* identified that 73% and 72% of the genes with correlated expression are induced after 30 min mite infestation for *NOI10* and *NOI11*, respectively. In contrast, only 28% of the most coexpressed genes with *RIN4* responded to the mite (Fig. 3A). Gene ontology analyses identified an enrichment of genes involved in common biological processes in the three sets of coexpressed genes (Supplementary Dataset S3). Networks of the most enriched biological processes for each set of genes showed enriched categories related to biotic responses (Fig. 3B–D).

Since the three genes participate in similar biological processes, they could share protein interactors. Physical interactors have been determined for RIN4 but not for NOI10 or NOI11. String-based network shows the functional and physical interactions predicted or experimentally determined for RIN4, including ten different proteins (Fig. 3E). Four of these proteins are encoded by genes upregulated after 30 min of mite infestation (Fig. 3F). Three of them, *RIPK*, *RPM1*, and *RPS2* encode receptor-like proteins and their interaction with RIN4 has been experimentally determined. These features prompted us to check if these proteins could also be interacting with NOI10 and NOI11. As the NOI proteins are anchored to the plasma membrane, a modified split-ubiquitin membrane Yeast Two-Hybrid (MYTH) screen was performed to check if RIPK, RPM1, and RPS2 can interact with the three two-NOI proteins. By this method, NOI10 and NOI11 proteins were found to interact with RIPK and RPS2 (Fig. 3G). In contrast, NOI3 and NOI5 showed no interaction with RIPK and were very weak with RPS2.

### NOI10 and NOI11 interact with RPM1 but have different effects than RIN4 on HR

**Fig. 4:**
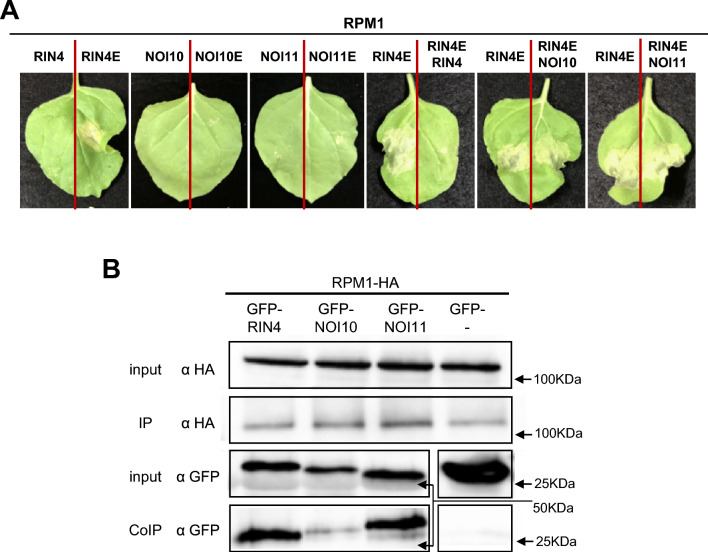
HR associated with RPM1-RIN4-like/NOIs interactions. (**A**) Cell death phenotype of *RPM1* and indicated RIN4-like/NOI combinations transiently expressed from the 35S promoter in *N. benthamiana*. (**B**) Co-immunoprecipitation assays of *N. benthamiana* extracts from leaves transiently expressing GFP-RIN4-like/NOIs and RPM1-HA under the control of the 35S promoter. The presence of proteins in the crude extracts and the immunoprecipitated fractions was determined by western blot analysis using anti-GFP and anti-HA antibodies. The experiments were independently repeated three times with similar results

In vitro interactions suggested an in vivo role for the NOI proteins in the RIN4-regulated mechanisms. Although positive results were not found in our MYTH assays, the interaction of RIN4 and RPM1 was previously reported (Mackey et al. 2002). Thus, RPM1 could also be an interactor of NOI10 and NOI11. Furthermore, the RIN4 phosphomimetic mutant RIN4^T166E^ (RIN4E) was able to induce HR in the presence of RPM1 when was transiently expressed in *N. benthamiana* leaves (Chung et al. 2011). Similar mutations were performed in the T166 equivalent residues of the NOI10 and NOI11 proteins, creating NOI10^T166E^ (NOI10E) and NOI11^T168E^ (NOI11E) mutants. To detect possible consequences on the HR, *N. benthamiana* plants were infiltrated with *A. tumefaciens* expressing different gene combinations. Macroscopic HR was not detected in leaves infiltrated with constructs expressing WT or RIN4E, NOI10E, and NOI11E mutant proteins alone (Supplementary Fig. S4). Confirming previous results, a slight RPM1 auto-activity was detected when the *RPM1* gene was expressed under the control of the 35S promoter (Prokchorchik et al. 2020) (Supplementary Fig. S4). This auto-activity was enhanced by the coexpression with RIN4E but not with NOI10E or NOI11E (Fig. 4A). Besides, RPM1 + RIN4E-induced HR was suppressed by the coexpression with RIN4, as previously observed (Xu et al. 2017), but not suppressed by the coexpression with NOI10 or NOI11 (Fig. 4A).

To test if these results could be explained by the different interacting capacities of RIN4, NOI10, and NOI11 proteins with RPM1, we performed co-immunoprecipitation (coIP) assays using proteins transiently expressed in *N. benthamiana*. Protein presence was checked by immunoblotting of the proteins RPM1-HA, GFP-NOI10, GFP-NOI11, and GFP-RIN4. CoIP assays demonstrated the in vivo interaction between RPM1 and the three tested proteins, RIN4, NOI10, and NOI11, although a weaker interaction with NOI10 was found, which supports the functional effects detected in the infiltration experiments (Fig. 4B).

### Interactions of NOI10, NOI11, and RIN4 with RIPK have similar effects on HR

**Fig. 5:**
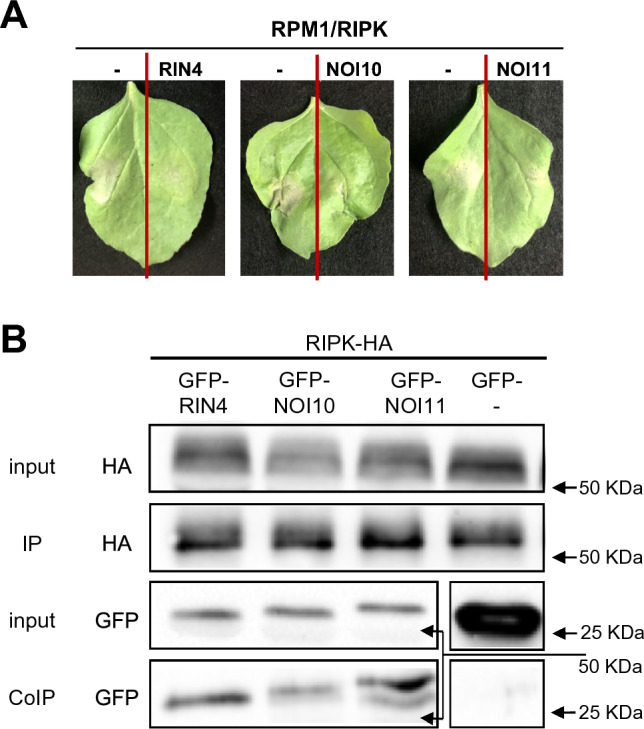
HR associated with RPM1-RIPK-RIN4-like/NOIs interactions. (**A**) Cell death phenotype of *RPM1*-*RIPK* and indicated RIN4/NOIs transiently expressed from the 35S promoter in *N. benthamiana*. (**B**) Co-immunoprecipitation assays of *N. benthamiana* extracts from leaves transiently expressing GFP-RIN4-like/NOIs and RPM1-HA under the control of the 35S promoter. The presence of proteins in the crude extracts and the immunoprecipitated fractions was determined by western blot analysis using anti-GFP and anti-HA antibodies. The experiments were independently repeated three times with similar results

RIPK is a receptor-like cytoplasmic kinase that can phosphorylate RIN4 at its T166 residue, phosphorylation necessary for RPM1 activation (Liu et al. 2011). As phosphomimetic mutants in the T166 position of NOI10, NOI11, and RIN4 cause different effects on RPM1-related HR, the interaction between NOI10 and NOI11 with RIPK, previously detected by MYTH assays, could also affect HR response. Whereas RIPK did not cause HR alone, the coexpression of RPM1 and RIPK in *N. benthamiana* leaves enhanced the HR response caused by RPM1 (Fig. 5A, Supplementary Fig. S4), suggesting that RIPK is activating RPM1-mediated HR. When RIN4, NOI10, or NOI11 were coexpressed with RPM1 and RIPK, a similar attenuation of the HR response was found (Fig. 5A).

To corroborate the protein–protein interactions detected by the MYTH technique, CoIP assays were performed using proteins transiently expressed in *N. benthamiana*. Immunoblotting of the protein extracts confirmed the presence of the proteins and CoIP assays demonstrated the RIN4, NOI10, and NOI11 interactions with RIPK (Fig. 5B).

### RPS2-induced HR is reduced by NOI10, NOI11, and RIN4

**Fig. 6:**
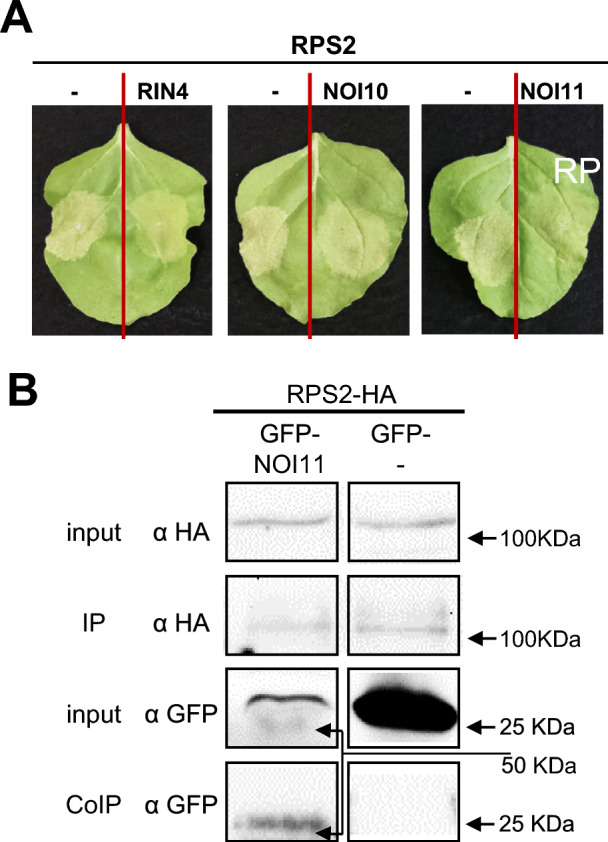
HR associated with RPS2-RIN4-like/NOIs interactions. (**A**) Cell death phenotype of *RPS2* and indicated RIN4-like/NOIs transiently expressed from the 35S promoter in *N. benthamiana*. (**B**) Co-immunoprecipitation assays of *N. benthamiana* extracts from leaves transiently expressing GFP-NOI11 and RPS2-HA under the control of the 35S promoter. The presence of proteins in the crude extracts and the immunoprecipitated fractions was determined by western blot analysis using anti-GFP and anti-HA antibodies. The experiments were independently repeated three times with similar results

The HR response associated with RPS2 activation depends on the presence of RIN4. Cleavage of RIN4 caused by the AvrRpt2 effector released RPS2 and triggered HR (Mackey et al. 2003). As NOI10 and NOI11 also interacted with RPS2 in our assays, their effect on the regulation of the RPS2-associated HR was checked. As expected, transient expression of RPS2 alone caused HR activation, and a slight reduction of HR was observed when RPS2 was coexpressed with RIN4, as reported previously (Day et al. 2005). When NOI10 or NOI11 were coinfiltrated with RPS2, similar reductions in the HR response were found (Fig. 6A).

Protein degradation occurred more rapidly after RPS2-induced HR, which made difficult the extraction of sufficient protein quantity for immunoblotting. In vivo interaction could only be demonstrated by CoIP for the NOI11-RPS2 proteins (Fig. 6B).

### NOI10 and NOI11 affect plant susceptibility to *P. syringae* pv tomato (*Pst*) DC3000 (AvrRpm1) and (AvrRpt2)

**Fig. 7:**
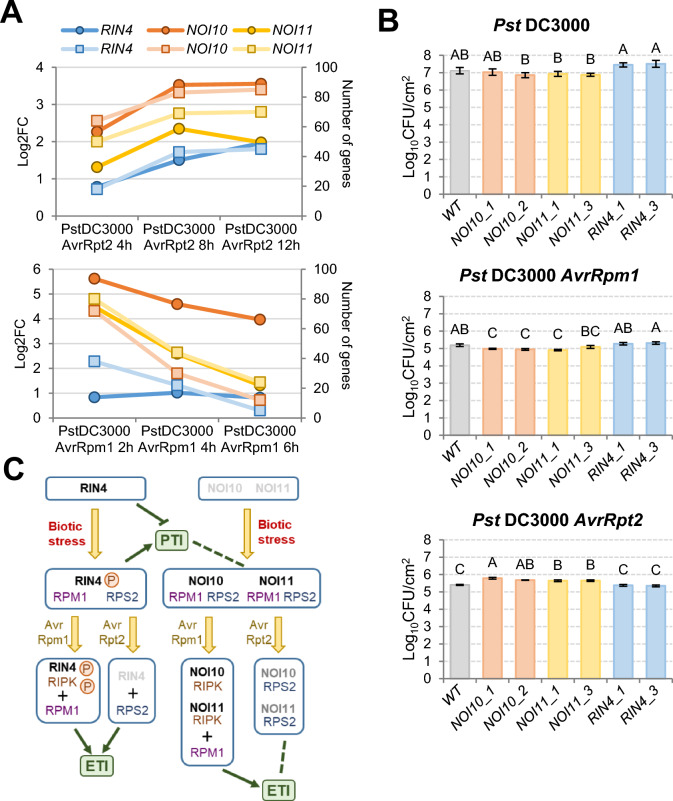
NOI10 and NOI11 affect resistance to bacteria. (**A**) Line graph showing the expression levels of *NOI10*, *NOI11*, and *RIN4* genes after *Pst* DC3000 (AvrRpt2) inoculation (left axis, circle markers, dark lines) and the number of the 100 most coexpressed genes for each gene that were induced by *Pst* DC3000 (AvrRpt2) (right axis, square markers, light lines). Data were obtained from the PlaD database. (**B**) Bacterial quantification after 24 h of leaf infiltration with different *Pst* DC3000 genotypes in WT and RIN4-like/NOI overexpressing lines. Data are representative of two independent experiments with similar results. Data are means ± SE of six biological replicates. Different letters indicate significant differences (*P* < 0.05, One-way ANOVA followed by Duncan multiple comparisons test). (**C**) Schematic model showing the participation of NOI10 and NOI11 in the RIN4-mediated defense responses. *PTI* pattern-triggered immunity, *ETI* effector-triggered immunity. Pointed head arrows indicate induction, blunt head arrow repression, and dashed lines no effect

Transient expression results suggest that NOI10 and NOI11 affect the defense responses regulated by RIN4. Therefore, NOI10 and NOI11 could be regulating the response of the plant to pathogen infections. Searches in transcriptomic databases showed an earlier upregulation of *NOI10* and *NOI11* transcript levels when Arabidopsis plants were challenged with *P. syringae* bacteria expressing the AvrRpt2 or the AvrRpm1 effectors (Fig. 7A). In addition, a higher number of genes induced by *Pst*DC3000 (AvrRpt2) or *Pst*DC3000 (AvrRpm1) were identified in the *NOI10* and *NOI11* lists of the 100 genes with the highest correlated expression than in the *RIN4* list (Fig. 7A). These results pointed to testing if the overexpression of the *NOI10*, *NOI11*, or *RIN4* genes affected the susceptibility of Arabidopsis plants to *Pst*DC3000 isolates expressing the AvrRpm1 or the AvrRpt2 effectors. CFU counting was used as an indicator of plant susceptibility. *NOI10* and *NOI11* overexpressing lines behaved similarly (Fig. 7B). These lines had lower susceptibility than WT plants to *Pst*DC3000 (AvrRpm1) and higher susceptibility to *Pst*DC3000 (AvrRpt2). In contrast, *RIN4* OE lines were more susceptible to *Pst*DC3000 (AvrRpm1) than WT plants and presented opposite effects on susceptibility than *NOI10* and *NOI11* OE lines. Their susceptibility was higher when plants were challenged with *Pst*DC3000 or *Pst*DC3000 (AvrRpm1) and lower against *Pst*DC3000 (AvrRpt2) (Fig. 7B). As expected, susceptibility to bacteria carrying any of the effectors was lower than that quantified to *Pst*DC3000.

## Discussion

The guard hypothesis establishes that R-proteins do not directly bind pathogen proteins but bind host proteins, named “guardees”, which are the direct targets of pathogen effectors (Jones and Dangl 2006). R-proteins perceive changes in their guardees and initiate a defense response. This hypothesis predicts that dissimilar effectors could bind the same guardee and that the changes caused in the guardee could activate more than one R protein, which has previously been experimentally confirmed for RIN4 (Yakura 2020). Our results add a new layer of complexity to this theory, with the existence of multiple related molecules acting as guardees or decoys involved in the response of shared guard R-proteins to common pathogen effectors. In addition to RIN4, NOI10, and NOI11 may act as guardees or decoys of the R-proteins RPM1 and RPS2 and as targets or decoys of the *P. syringae* effectors AvrRpm1 and AvrRpt2.

Several pieces of evidence suggest that the functionality of NOI10 and NOI11 as putative guardees or decoys differs from that observed for RIN4. A major difference between them is their basal expression and their induction by biotic treatments. RIN4 is constitutively expressed as expected for a guardee protein. In unchallenged states, RIN4 binds the R-proteins RPM1 and RPS2, maintaining R-proteins in an inactive state (Kim et al. 2005). Upon infection with *P. syringae* strains that produce the AvrRpm1 or AvrB effectors, hyperphosphorylation of RIN4 by RIPK or other kinases occurs (Chung et al. 2011; Liu et al. 2011; Mackey et al. 2002). The phosphorylation of RIN4 in T166 activates RPM1-mediated ETI, triggering downstream signaling events and HR. In the presence of the AvrRpt2 effector, RIN4 is proteolytically cleaved, which activates RPS2-mediated ETI (Axtell and Staskawicz 2003; Mackey et al. 2003). Conversely to RIN4, the basal expression of NOI10 and NOI11 is extremely low, and their hypothetical function as classical guardee proteins is unlikely. However, we have demonstrated that NOI10 and NOI11 also bind the R-proteins RPM1 and RPS2, as well as the kinase RIPK. Besides, the cleavage of NOI10 and NOI11 proteins by AvrRpt2 effector was previously reported (Eschen-Lippold et al. 2016) and AvrRpm1 associates with and ADP ribosylates most Arabidopsis RIN4-like/NOI proteins, including NOI11 (Redditt et al. 2019).

We wondered then why these RIN4-like/NOI genes differ in their transcriptional regulation. Two possibilities arise, they could reinforce the role of RIN4 or they could hinder the functionality of RIN4. Several shreds of evidence support the inexistence of an obvious synergism of NOI10 or NOI11 in RIN4 activation. First, the phosphomimetic forms NOI10E and NOI11E affecting the key residue threonine 166 did not cause HR in *N. benthamiana* leaves when coexpressed with RPM1. Threonine 166 phosphorylation depends on the ADP-ribosylation of D153, and that RPM1-mediated defense response is enhanced by the phosphorylation of T21 and S160 (Liu et al. 2011; Redditt et al. 2019). As previously reported, equivalent residues to RIN4 D153 and S160 are not present in NOI10 and NOI11 (Contreras and Martinez 2022). Therefore, phosphorylation of T166 in NOI10 and NOI11 could not occur at all or have different functional consequences than that reported for RIN4. A second piece of evidence comes from the inability of NOI10 and NOI11 to avoid HR in leaves coinfiltrated with RPM1 and RIN4E, the phosphomimetic of RIN4 phosphorylated in T166. Effective HR suppression was found when unphosphorylated RIN4 was coexpressed with the combination of RPM1 and RINE, confirming previous results (Xu et al. 2017). Besides, RIN4 as well as NOI10 and NOI11 diminished the HR triggered by the coexpression of RPM1 and RIPK in *N. benthamiana*, our data supported that the HR decrease could be due to different mechanisms. The response to RIN4 would be an equilibrium between the blocking of the defense response caused by the binding of RIN4 to RPM1 and the triggering of the HR due to the phosphorylation of RIN4 by RIPK. In contrast, NOI10 and NOI11 would reduce HR by competing for RIPK binding. Although RIPK could phosphorylate NOI10 and NOI11, the RPM1-mediated HR would not be triggered as it was observed for the coexpression of RPM1 with the NOI10E and NOI11E mutants.

As *NOI10* and *NOI11* are upregulated after mite treatment, they should be involved in the induced defense response of the plant. Although the overexpression of *NOI10* and *NOI11* did not cause reduced damage in the leaves, their expression correlated with the expression of many genes also upregulated after *T. urticae* infestation. The overexpression of the mite-induced *NOI3* and *NOI5*, which encode single-NOI proteins, neither significantly changed leaf damage. Thus, the role of the RIN4-like/NOI proteins should be considered in the context of the induction of general defense mechanisms and not as specific in the plant–mite interaction. Overexpressing *NOI10* and *NOI11* plants have significant differences in *P. syringae* susceptibility with WT and OE *RIN4* plants. It was described that *Pst*DC3000 expressing the AvrRpm1 or AvrRpt2 effectors grew slower than *Pst*DC3000 lacking these effectors in Arabidopsis (Mackey et al. 2002). In addition, *Pst*DC3000 and *Pst*DC3000 (AvrRpm1) grew similar in WT plants and in plants overexpressing *RIN4*, and *Pst*DC3000 (AvrRpt2) grew better in overexpressing *RIN4* plants (Mackey et al. 2002). In general, these results were confirmed in our study. Additionally, we found that OE *NOI10* and *NOI11* lines were more resistant to *Pst*DC3000 (AvrRpm1) and more susceptible to *Pst*DC3000 (AvrRpt2) than WT and OE *RIN4* plants. These findings further support a different role of NOI10/NOI11 and RIN4 in the control of RPM1 and RPS2 signaling, which is reinforced by the coexpression of *NOI10*, *NOI11*, and *RIN4* with dissimilar sets of defense-related genes.

The higher resistance of OE *NOI10* and *NOI11* plants to *Pst*DC3000 (AvrRpm1) has not a straightforward explanation. Our data shows that, although NOI10 and NOI11 produced HR attenuation in *N. benthamiana* leaves coexpressing RPM1 + RIPK and the phosphomimetic NOI10E and NOI11E did not trigger HR, NOI10 and NOI11 did not suppress RPM1 + RIN4E-mediated HR. Therefore, we hypothesize that NOI10 and NOI11 could affect the RIN4-mediated pathway by competing for RIPK binding, but with specific features leading to a more efficient defense. The results obtained about the control of RPS2-mediated defense have a clearer interpretation. As AvrRpt2 cleavage of RIN4 leads to RPS2 activation, we can hypothesize that NOI10 and NOI11 bind RPS2 and reduce the HR response caused by RPS2 in *N. benthamiana* leaves in a similar way to the reduction observed when RPS2 is coexpressed with RIN4 (Day et al. 2005; Kim et al. 2005). Therefore, the induction of NOI10 and NOI11 could reinforce the basal role of RIN4 in blocking the RPS2-mediated response, leading to an exacerbated susceptibility. Besides, *Pst*DC3000 (AvrRpt2) bacteria grew better in plants overexpressing *NOI10* or *NOI11* than in OE *RIN4* plants, which suggests a stronger inhibition of RPS2 activity in these lines. The differences in the amino acid sequence and structure of the two-NOI proteins can explain this behavior. NOI10 and NOI11 do not have the conserved AvrRpt2 cleavage site in the C-NOI domain (Contreras and Martinez 2022). Thus, a more stable interaction with RPS2 is expected, which would lead to a minor activation of the RPS2-mediated defense response.

All this information should be considered in the context of the PTI and ETI defense responses (Fig. 7C). RIN4 is considered a negative regulator of both PTI and ETI responses in unchallenged plants (Zhao et al. 2021). Pathogen recognition by PRRs leads to posttranslational modification of RIN4 activating PTI responses. Further RIN4 modifications caused by bacterial effectors hindered PTI-triggered and activate NLR-mediated ETI responses. Recent studies highlighted the convergence of these pathways on largely similar responses and established the mutual requirement and potentiation of PTI and ETI to reach an optimal immune response (Ngou et al. 2021; Tian et al. 2021; Yuan et al. 2021). Therefore, the role of NOI10 and NOI11 should reflect the interconnections between PRR and NLR-induced pathways. Earlier immune responses include callose accumulation (Wang et al. 2021). After mite infestation, callose accumulation is restricted by overexpression of *RIN4*, but not of *NOI10* and *NOI11*, which suggests that NOI10 and NOI11 did not have a negative effect on PTI. Besides, NOI10 and NOI11 did not compete with RIN4E as RIN4 did in the establishment of HR, which could indicate a positive effect on ETI. However, NOI10 and NOI11 have antagonistic effects in defense against *Pst*DC3000 (AvrRpm1) and *Pst*DC3000 (AvrRpt2). It has been reported that about 50% of regulated genes from the RPS2-mediated transcriptional response overlapped with the RPM1-mediated transcriptional response (Mine et al. 2018), while about 80% of regulated genes overlapped in the translational response (Meteignier et al. 2017; Yoo et al. 2020). These results suggest that not only translational changes during ETI are likely a general pattern but also point to unequal effects on gene expression depending on the effector molecule. Thus, the modulation of the defense response caused by NOI10 and NOI11 would be the consequence of their individual effects in both, the PTI and the ETI responses.

In conclusion, the participation of proteins of the RIN4-like/NOI family other than RIN4 in Arabidopsis immunity has been demonstrated. Upregulation of *NOI10* and *NOI11* modulates the immune responses triggered by the activation of R-proteins upon the modification of its guardee RIN4 protein. A role as decoys could be exerted by NOI10 and NOI11 in the connecting pathway between common effectors and shared guard R-proteins. In this way, NOI10 and NOI11 would help to integrate external signals in a more efficient response.

### Supplementary Information

Below is the link to the electronic supplementary material.

Supplementary file1 (PDF 315 kb)

Supplementary file2 (XLSX 13 kb) Sequences of the oligonucleotides used in this work

Supplementary file3 (XLSX 40 kb) Lists of the 100 most significant coexpressed genes with *NOI10*, *NOI11*, and* RIN4*

Supplementary file4 (XLSX 22 kb) Lists of the enriched biological processes using the 100 most coexpressed genes with *NOI10*, *NOI11*, and *RIN4*

## Data Availability

All data generated or analyzed during the present study can be found within the manuscript and its supporting materials.
