# Supplementary material for: The RIN4-like/NOI proteins NOI10 and NOI11 modulate the response to biotic stresses mediated by RIN4 in Arabidopsis
Source: Plant Cell Rep. 2024 Feb 15;43(3):70. doi: 10.1007/s00299-024-03151-9 (PMC10869442; doi:10.1007/s00299-024-03151-9)
Supplement: Supplementary file 1 — Supplementary file1 (PDF 315 kb) [file 299_2024_3151_MOESM1_ESM.pdf]

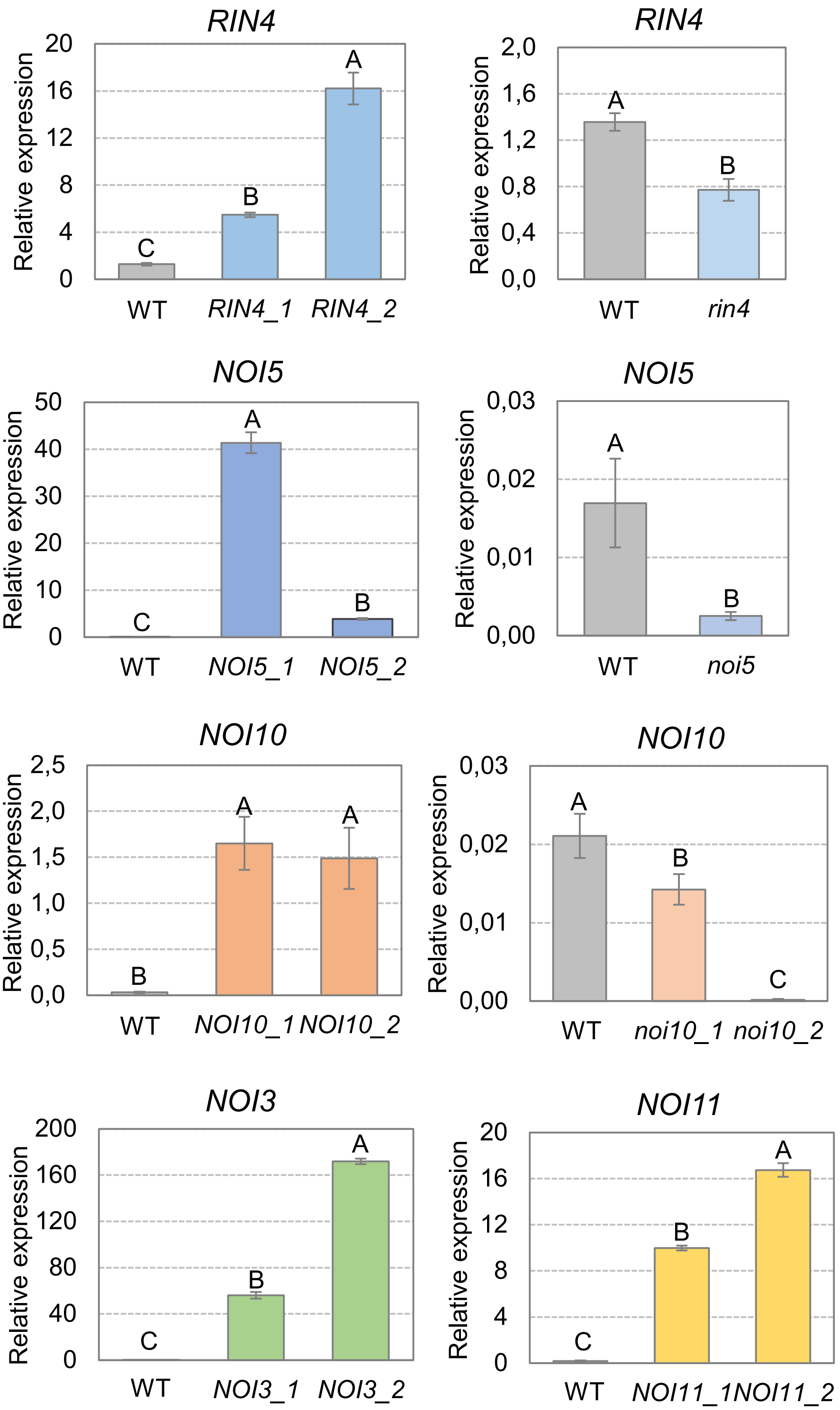

Supplementary Figure S1. Relative expression levels of *NOI3*, *NOI5*, *NOI 10*, *NOI11*, and *RIN4* in WT, overexpressing, and T-DNA inserted lines. Data are means ± SE of three biological replicates. Different letters indicate significant differences (P<0.05, One-way ANOVA followed by Duncan multiple comparisons test).

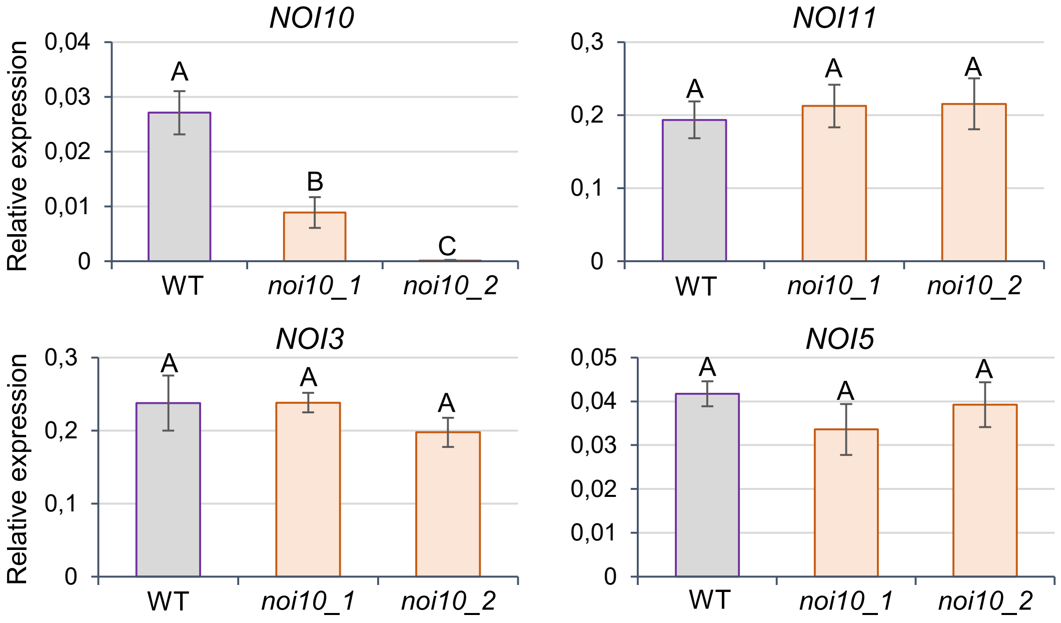

Supplementary Figure S2. Relative expression levels of *NOI3*, *NOI5*, *NOI 10*, and *NOI11* in WT and *noi10* T-DNA inserted lines. Data are means ± SE of three biological replicates. Different letters indicate significant differences (P<0.05, One-way ANOVA followed by Duncan multiple comparisons test).

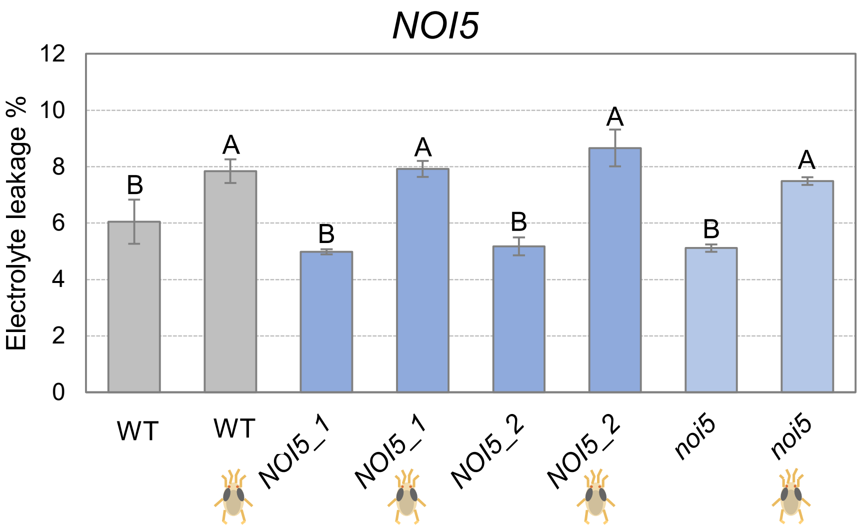

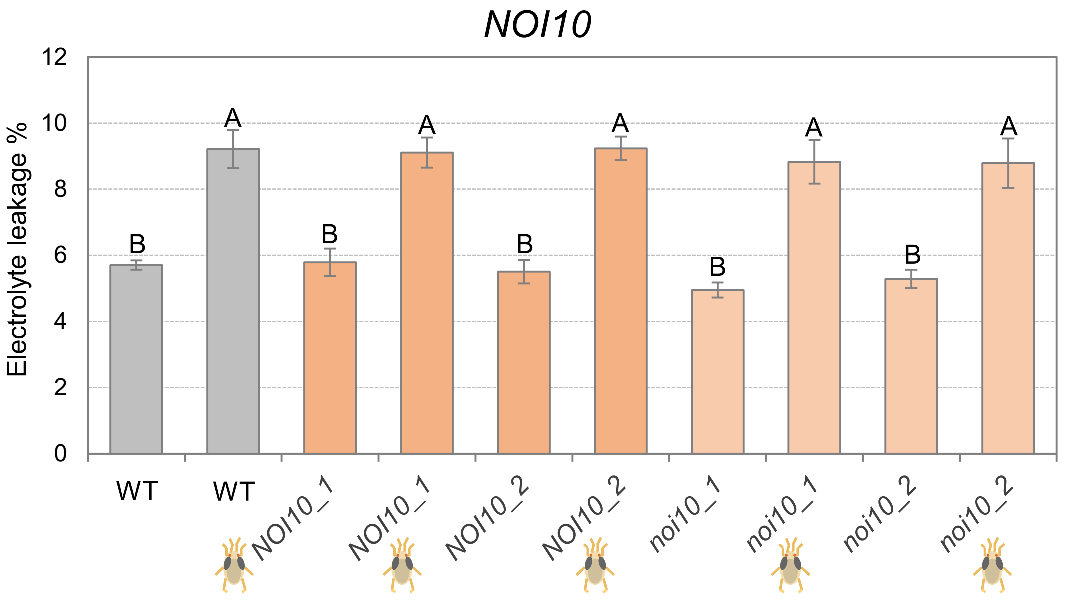

Supplementary Figure S3. Electrolyte leakage in WT, *NOI5*, and *NOI10* genotypes before and after *T. urticae* infestation. Electrolyte leakage into deionized water was measured as changes in conductivity and the percentages shown are means ± SE of three biological replicates. Different letters indicate significant differences (P<0.05, One-way ANOVA followed by Duncan multiple comparisons test).

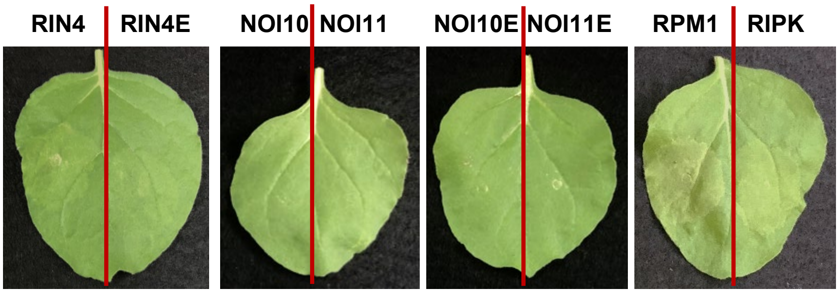

Supplementary Figure S4. HR phenotypes associated with single agroinfiltrations. Cell death phenotype of *RPM1*, *RIPK*, and indicated RIN4-like/NOIs transiently expressed from the 35S promoter in *N. benthamiana*.
